# Distinct climate influences on the risk of typhoid compared to invasive non-typhoid *Salmonella* disease in Blantyre, Malawi

**DOI:** 10.1038/s41598-019-56688-1

**Published:** 2019-12-30

**Authors:** Deus Thindwa, Michael G. Chipeta, Marc Y. R. Henrion, Melita A. Gordon

**Affiliations:** 1 Malawi-Liverpool-Wellcome Trust Clinical Research Programme, Blantyre, Malawi; 2 Centre for Mathematical Modelling of Infectious Diseases, Department of Infectious Disease Epidemiology, London School of Hygiene and Tropical Medicine, London, United Kingdom; 3 Nuffield Department of Medicine, Big Data Institute, Oxford University, Oxford, United Kingdom; 4 Department of Clinical Sciences, Liverpool School of Tropical Medicine, Liverpool, United Kingdom; 5 Institute of Infection and Global Health, University of Liverpool, Liverpool, United Kingdom; 6 Malawi College of Medicine, University of Malawi, Blantyre, Malawi

**Keywords:** Environmental health, Bacterial infection, Statistics

## Abstract

Invasive *Salmonella* diseases, both typhoid and invasive non-typhoidal *Salmonella* (iNTS), are seasonal bloodstream infections causing important morbidity and mortality globally in Africa. The reservoirs and transmission of both are not fully understood. We hypothesised that differences in the time-lagged relationships of rainfall or temperature with typhoid and iNTS incidence might infer differences in epidemiology. We assessed the dynamics of invasive *Salmonella* incidence over a 16-year period of surveillance, quantifying incidence peaks, seasonal variations, and nonlinear effects of rainfall and temperature exposures on the relative risks of typhoid and iNTS, using monthly lags. An increased relative risk of iNTS incidence was short-lasting but immediate after the onset of the rains, whereas that of typhoid was long-lasting but with a two months delayed start, implying a possible difference in transmission. The relative-risk function of temperature for typhoid was bimodal, with higher risk at both lower (with a 1 month lag) and higher (with a ≥4 months lag) temperatures, possibly reflecting the known patterns of short and long cycle typhoid transmission. In contrast, the relative-risk of iNTS was only increased at lower temperatures, suggesting distinct transmission mechanisms. Environmental and sanitation control strategies may be different for iNTS compared to typhoid disease.

## Introduction

Invasive *Salmonella* diseases, both typhoid and invasive non-typhoidal *Salmonella* (iNTS) disease, are serious bloodstream infections co-existing in Africa, and are leading causes of morbidity and mortality worldwide. Typhoid, caused by serotype *S*. Typhi^1–3^, affects mainly healthy children and young adults, has a 1–2% case fatality, and is estimated to cause 9.9–24.2 million cases and 75,000–208,000 deaths globally per year^4–6^. On the other hand, iNTS disease in Africa is predominantly caused by non-typhoidal serovars *S*. Typhimurium and *S*. Enteritidis^7^. These serovars typically cause diarrhoeal disease among immunocompetent individuals in high income settings, which is often self-limiting^7^. In Africa, however, they cause severe NTS disease in adults with HIV and very young susceptible children with HIV, malaria or malnutrition^8^. iNTS disease carries a case-fatality of 10–20% in both adults and children, 10-fold higher than typhoid^9,10^, and leads to an estimated 2.1–6.5 million cases and 415,164–1,301,520 deaths globally per year^11,12^.

Typhoid occupies a human-restricted reservoir and is transmitted primarily through the faeco-oral route, either by individuals shedding bacteria in stool during acute or sub-acute illness, or by long-term asymptomatic human carriers^2,13^. The relative importance of these two human sources of transmission is likely to vary in different settings. Typhoid has a complex pattern of transmission cycles, involving a direct within-household “short cycle” and an environmental “long cycle”, involving contaminated water sources^13^. The reservoir and transmission routes of iNTS disease, in contrast, remain uncertain. NTS typically have a broad vertebrate host-range. In industrialised countries, where invasive disease is uncommon, NTS diarrhoeal disease is considered as a food-borne zoonosis, linked to industrialised food animal production. But in Africa, in a setting where there is a large population of immunologically susceptible individuals, there is growing evidence that humans may provide the reservoir and/or transmission routes for the NTS strains that cause invasive disease, since household case-control studies show no genomic overlap with animal-related NTS strains^14,15^.

Malawi experienced successive outbreaks of multi-drug resistant strains of *S*. Enteritidis, *S*. Typhimurium and *S*. Typhi which peaked in 2002–03, 2004–5 and 2013–14, respectively^13,16–19^. The emergence of resistance to chloramphenicol, ampicillin and co-trimoxazole meant that antibiotic use shifted to 3^rd^ generation injectable cephalosporins and/or oral fluoroquinolones, particularly ciprofloxacin for treatment. A cost-effective typhoid conjugate vaccine is in clinical trials in Africa and it is expected to reduce the disease burden especially among infants and school-age children^20–23^, and may also reduce broad spectrum antimicrobial usage. By contrast, new vaccines for NTS remain in early pre-clinical development, but understanding epidemiological and serological data will be essential for the design and assessment of iNTS vaccine trials’ impact through surveillance^11,24,25^.

Rainfall and temperature have been reported to increase *Salmonellae* abundance, water and food poisoning, inhibit host immunity, disrupt health systems by flooding, and increase malnutrition linked to droughts or floods^2,3,8,26–28^. Globally, some studies have reported differences in the effects of climate on the risk of *Salmonella*, suggesting setting-specific infection dynamics^13,29–34^. However, much is unknown about delayed structures of climate dynamics and invasive *Salmonella* disease, and how they relate to reservoirs and transmission routes, in poor resource settings. Moreover, climates may mediate their effects on disease not only through contamination of the household or environment, but also a seasonal influence on predisposing conditions, such as malaria (by mosquito breeding cycles) or malnutrition (through crops and food security)^8^. A comparison of time-lagged effects of climate dynamics on typhoid versus iNTS incidences has not been conducted. Malawi has, uniquely, a known high incidence of both typhoid and iNTS disease, and uninterrupted and consistent longitudinal blood culture surveillance of typhoid and iNTS for 20 years, allowing the current comprehensive analysis.

With this background, we studied climate in relation to *Salmonella* incidence prior to vaccine introduction in Malawi. Understanding how climate influences *Salmonellae* transmission may guide financial investments in preventive and curative interventions^35^, reformulate food/water security policies^32,36^, promote a comprehensive approach to climate risk preparedness^32,35,36^, and influence vaccination and surveillance programmes.

## Methods

### Study area

**Figure 1:**
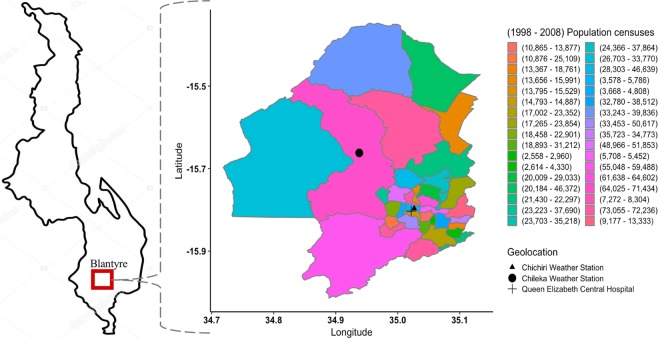
The map of Blantyre, Malawi. Shows locations by longitude and latitude of the weather stations (Chichiri and Chileka) which captured daily rainfall and temperature, Queen Elizabeth Central Hospital where invasive nontyphoid and typhoid cases were diagnosed, and the total population enumerated during 1998 and 2008 national censuses in Blantyre.

We conducted the study in Blantyre district, the commercial capital of Malawi, located at 35° east of Greenwich Meridian and 15° 42″ south of the Equator, and at an elevation of 1,041 meters above sea level^37^. Blantyre spans 2,012 km^2^ and is administratively split into 25 traditional authority boundaries with a total population of approximately 1,171,978 in 2015^38^, with a higher population density in its urban (3,006/km^2^) than peri-urban (190/km^2^) areas. Blantyre is, thus, representative of many urban centres in the country^38^. All febrile illnesses from invasive *Salmonella* infections in this analysis were diagnosed and treated at the Queen Elizabeth Central Hospital (QECH), the provider of referral and primary free healthcare to Blantyre (peri-) urban population as well as southern Malawi (Fig. 1).

### Laboratory surveillance data

QECH, through the Malawi-Liverpool-Wellcome Trust Clinical Research Programme (MLW), maintains a surveillance database of invasive *Salmonella* and other blood stream bacterial infections dating back to the late 1990s^13,39^. In the current study, we obtained the blood samples from febrile children and adults attending QECH, in whom *Salmonella* infections were suspected. The criteria for blood culture, and the laboratory blood-culture procedure used to isolate *Salmonella* from samples have essentially been consistent throughout this period, and have been described elsewhere^8,40,41^. In our analysis, we classified *S*. Enteritidis, *S*. Typhimurium and other non-*S*. Typhi *Salmonella* isolates as iNTS, and all *S*. Typhi isolates as typhoid. Using 1998 and 2008 Malawi census data, we estimated yearly populations during the study period from 2000 to 2015 by linear interpolation and extrapolation^38^. To visually show seasonality in observed *Salmonella* cases, we decomposed and seasonal-adjusted cases time series, and estimated minimum monthly incidence of iNTS, and typhoid per 100,000 population using Eq. (1) (Supplementary Figs. S1 and S2).

1 $$Incidence=\frac{\,{\rm{monthly}}\,{\rm{cases}}}{{\rm{middle}}\,{\rm{year}}\,{\rm{population}}}\ast 100,000$$

### Meteorological data

Malawi’s climate has unique rainy (November through April) and dry (May through October) seasons. However, the dry season is subdivided into cool (May through August) and hot (September through October) periods^42^. The early (November through February) and late (March through April) rainy seasons are dominated by tropical and extra tropical influences, respectively, and the interannual variability in these two periods is uncorrelated^43^. Typically, Malawi’s annual total rainfall varies between 725 and 2,500 mm, with Blantyre receiving 1,127 mm resulting in high intensity with heavy surface runoff at the beginning of the season^44,45^. The minimum and maximum daily temperatures of 14 and 43 °C are common in July and October, respectively^45^. In our analysis, we defined the average monthly rainfall (or temperature) as daily average rainfall (or temperature) between Chichiri and Chileka stations, and aggregated by month of the calendar year using lubridate R package^46^. We, similarly, showed the seasonality in rainfall and temperature values through their decomposed and seasonal-adjusted time series (Supplementary Figs. S1 and S2).

### Modelling framework

We fitted generalized linear models^47^ to the incidence of iNTS per month over 11 years, from January 2000 to December 2010, and of typhoid per month over 5 years, from January 2011 to December 2015. The seasonal-unadjusted incident cases of iNTS or typhoid on month *t* (*Y*_*t*_) was assumed to follow an overdispersed Poisson distribution with mean (*λ*_*t*_) and variance (*ϕλ*_*t*_), where *ϕ* is an estimated overdispersion parameter. The general mathematical form of the model fitted to the time series data is given by Eq. (2), following the standard guidelines^48^.

2 $$\begin{array}{c}{Y}_{t} \sim Poisson({\lambda }_{t},\phi )\\ \,Log({\lambda }_{t})=\alpha +{\beta }_{{t}^{\text{'}}}+{\mu }_{T}+f.w({x}_{1t},l)+f.w({x}_{2t},l)+{\varepsilon }_{t}\end{array}$$

where *λ*_*t*_ *E*(*Y*_*t*_) is the mean number of incident cases of iNTS or typhoid for month *t* where *t* = 1, …, 132 (11 years for iNTS) and *t* = 1, …, 60 (5 years for typhoid), *α* is the model intercept, ${\beta }_{{t}^{\text{'}}}$ are ‘months’ random effects to account for seasonality where, *t*′ = 1, …, 12 indexes the calendar month, *μ*_*T*_ are ‘years’ random effects to account for unmeasured interannual variability where T = 1, …, 5 (for typhoid) and T = 1, …, 11 (for iNTS) indexes the year, the cross-basis functions *f*.*w*(*x*_1*t*_,*l*) and *f*.*w*(*x*_2*t*_,*l*) are the nonlinear rainfall-lag and temperature-lag natural cubic spline functions, respectively, with lags *l* from 0 to 8 months, *ε*_*t*_ are the residuals added at specific lags to correct for partial autocorrelation.

**Figure 2:**
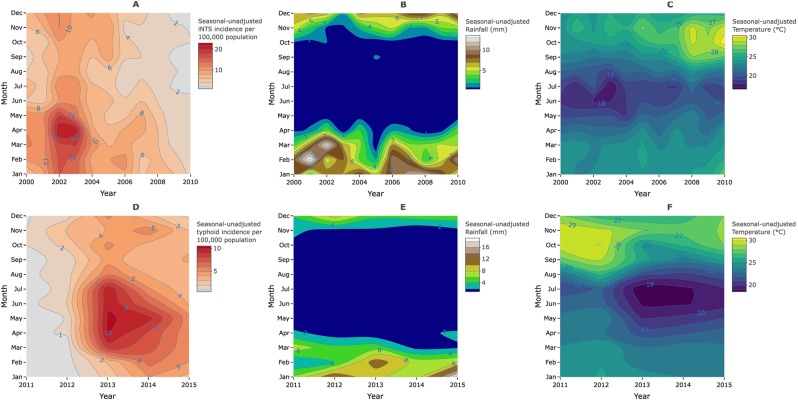
Seasonal dynamics of invasive *Salmonella* diseases. Contour plots of yearly and monthly changes in the incidence of nontyphoid *Salmonella* disease (iNTS), rainfall and temperature respectively (**A**–**C**) from 2000–2010; yearly and monthly changes in the incidence of typhoid, rainfall and temperature respectively (**D**–**F**) from 2011 to 2015 in Blantyre, Malawi.

Our study aimed to determine the seasonal and deseasonalized fluctuations of invasive *Salmonella* diseases that could inform the timing of possible control measures. Hence, we focused on data from the time periods that enabled us to do so. The iNTS data after 2010, and typhoid data before 2011 are outside of the major outbreak years, and were thus excluded from the analysis. (Fig. 2 and Supplementary Fig. S3). We apply the distributed lag non-linear modelling framework (DLNM) using the “dlnm” R package^49^, to simultaneously investigate the potential nonlinear and delayed effects of rainfall and temperature on monthly iNTS or typhoid incidence. We captured rainfall, temperature and lags using natural cubic spline functions to flexibly model nonlinear climate-lag structures and their relationships on the risk of invasive *Salmonella* diseases (typhoid and iNTS). These combinations yielded cross-basis matrices of rainfall-lag and temperature-lag. Variables for seasonality, interannual variability, rainfall-lags, temperature-lags and residuals were all included as terms in the overdispersed Poisson regression model to estimate the relative risk of iNTS. On the other hand, we fitted two separate models to estimate the relative risk of typhoid. Both models included variables for seasonality and interannual variability in addition to rainfall-lag and residuals for the first model, and rainfall-lag and temperature-lag for the second model.

### Model selection, evaluation and estimation

We generated a total of 162 potential models from a combination of degrees of freedom (*df*) representing each climate-lag spline function. A natural cubic spline is linear beyond the boundary knots thereby imposing at least 2 knots. Additional knots within the boundary interval in the lag/exposure spaces implies (*m* + 1) degrees of freedom per year where *m* is the number of knots (assuming no intercept). We imposed 2 internal knots (3 *df* per year), 3 (4 *df* per year) or 4 (5 *df* per year) without an intercept to model the effects of rainfall, temperature and lag simultaneously. Cross-basis matrices resulting from the combinations of the *df* of the lag and climate variables were included in overdispersed Poisson model as shown in Eq. (2). We selected the models that minimized the Quasi-Akaike Information Criterion (Q-AIC), which is asymptotically equivalent to a cross-validation statistic^50^. The Q-AIC values were calculated using Eq. (3), and the results are shown in Supplementary Table S1. In addition to selecting the best fitting models using Q-AIC, we examined the residual deviances overtime, autocorrelation and partial autocorrelation of the selected models. If the model (termed ‘original model’) showed significant partial autocorrelation at specific lags, residuals at those lags were added to the model (now termed the ‘adjusted model’) in order to reduce partial autocorrelation to below the significant threshold shown by blue dotted lines (Supplementary Fig. S4). The final model estimates were calculated from the ‘original’ or ‘adjusted’ models using quasi-maximum likelihood estimator which is assumed to be consistent and asymptotically normally distributed regardless of the cases generation process.

3 $$QAIC=-2L(\theta )+2\phi k$$

where *L* is the log-likelihood of the Poisson distribution fitted model with parameter *θ*, *ϕ* is the estimated overdispersion parameter and *k* is the number of model parameters^51^.

In a model selection sensitivity analysis, we assessed the impact of varying the *df* in the cross-basis functions of our final models on estimated relative risks of invasive *Salmonella* diseases (Supplementary Figs. S5 and S6). In summary, our final relative risk estimates are based on the final models with the following choices; (1) monthly random effects to control for seasonality, (2) yearly random effects to control for long-term trends and interannual variability, (3a) cross-basis matrices for {rainfall, lag} with {3, 3} *df* per year and {temperature, lag} with {3, 3} *df* per year to estimate the effects of rainfall and temperature on relative risk of iNTS, (3b) cross-basis matrix for {rainfall, lag} with {4, 3} *df* per year to estimate the effects of rainfall on relative risk of typhoid, and (3c) cross-basis matrices for {rainfall, lag} and {temperature, lag} each with {3, 3} *df* per year to estimate the effects of temperature on relative risk of typhoid (Supplementary Table 1). Natural spline functions were chosen to allow for more parsimonious models^52^. Selection of monthly-scale lag and maximum lag of up to 8 months was based on observation of much lower invasive *Salmonella* diseases incidence on weekly scale with insignificant disease dynamics (Supplementary Figs. S7 and S8), and knowledge from previous mathematical model that showed that typhoid incidence was highly correlated with rainfall at lags 6–21 weeks^13^, hence, a need to capture important association at longer lags without loss of precision in estimates. Moreover, the heatmaps from using weekly-scale data of up to 5 weeks maximum lag clearly showed significant uncontrolled residual autocorrelation resulting in unclear patterns of increased or decreased risk of invasive *Salmonella* diseases (Supplementary Fig. S9). All analyses were conducted in R v3.2.4^53^. Statistical significance was p < 0.05.

### Ethical approval

*Salmonella* isolates described in this study were obtained from febrile Malawian adults and children as part of the routine case management when they attended QECH. The use of routine clinical case management samples was granted by the College of Medicine Research Ethics Committee (COMREC) under approval P.08/14/1614, in compliance with Malawi Government regulations, through the National Commission for Science and Technology (NCST). Individual patient informed consent was not required for the use of publicly available anonymised routine samples as per COMREC guideline 5.6. Climate data was obtained with permission from the Department of Climate Change and Meteorological Services in Blantyre, Malawi.

## Results

### Descriptive analysis

**Figure 3:**
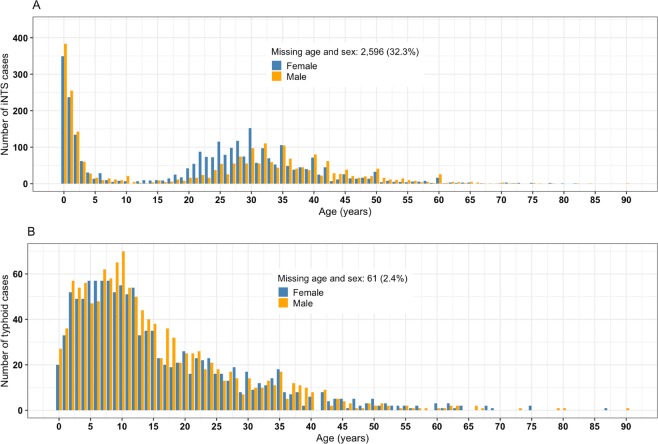
Age and sex distribution of invasive *Salmonella* cases. Nontyphoid (iNTS) (**A**) and typhoid (**B**) cases confirmed by blood culture at the Malawi Liverpool Wellcome Trust Clinical Programme in Blantyre, Malawi over the yearly period from 2000 to 2015.

Of the 12,166 patients with confirmed invasive *Salmonella* diseases at QECH from 2000–2015, 9,518 (78.2%) were iNTS disease cases of *S*. Enteritidis (15%, n = 1,423), *S*. Typhimurium (80%, n = 7,611) and other non-*S*. Typhi isolates (5%, n = 484), and 4,259 (48.4%, n = 8,797) were males. iNTS cases had a median age of 7 years (interquartile range [IQR]: 1–31; n = 8,025), and typhoid cases of 12 years (IQR: 6.4–22; n = 2,529) (Fig. 3). During the 11-years of iNTS and 5-years of typhoid surveillance included in this analysis, the mean minimum incidence rates of iNTS and typhoid were 7.1, 95%CI (6.3–7.8) and 3.6, 95%CI (2.9–4.4) per 100,000 population, respectively, at the main hospital. iNTS disease incidence increased from year 2000 with a peak in 2002–2003 (16 cases per 100,000 population), but subsequently dropped year-on-year for the next decade. On the other hand, typhoid had not emerged until 2011 with an incidence peak during the 2013–2014 outbreak (11 cases per 100,000 population) (Fig. 2).

### Comparing the delayed effects of rainfall on iNTS and typhoid

**Figure 4:**
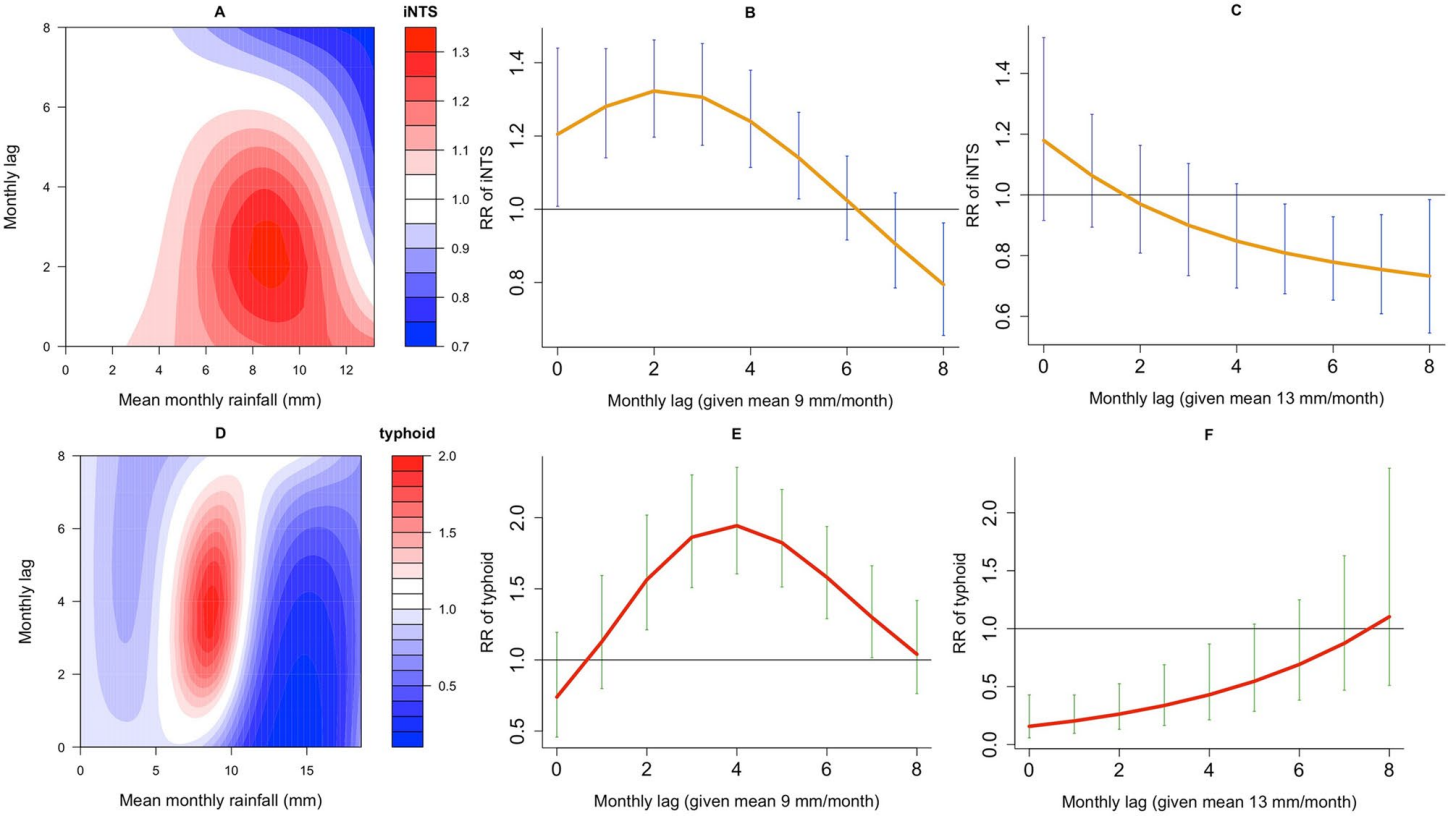
Relative risk (RR) of invasive *Salmonella* diseases given rainfall exposures and monthly lags. Contour plot of rainfall-lag-nontyphoid (iNTS) (**A**) relative to baseline mean monthly rainfall conditions of 0 mm at lags between 0 and 8 months, and lag-iNTS curve plots at 9 mm (**B**) and 13 mm (**C**) of rainfall with 95% Confidence Intervals; Contour plot of rainfall-lag-typhoid (**D**) relative to baseline mean monthly rainfall conditions of 0 mm at lags between 0 and 8 months, and lag-typhoid curve plots at 9 mm (**E**) and 13 mm (**F**) of rainfall with 95% Confidence Intervals.

Figure 4 summarises the modelled rainfall-lag relationships for both iNTS and typhoid diseases through contour and curve plots of the estimated relative risks (RR) along rainfall and lags compared to a reference mean monthly rainfall of 0 mm. A 9 mm rainfall-lag relationship for iNTS seems to vary with lag, with immediate and significant increased effect from lags 0 to 5 months, reaching the maximum effect at lag 2 (RR 1.32, 95%CI [1.20–1.46]), and with delayed and significant reduced effect at lag 8 (RR 0.79, 95%CI [0.66–0.96]). Comparatively, under the same reference value, the rainfall-lag relationship for typhoid depicts a delayed and significant increased effect of 9 mm of rainfall on the risk of typhoid from lags 2 to 7 months, with the maximum effect at lag 4 (RR 1.94, 95%CI [1.60–2.35]). Moreover, the effects of excessive rainfall (≥13 mm) are associated with significant reductions in both iNTS and typhoid at many lags (Fig. 4 and Table 1).

**Table 1:** Relative risk (RR) of invasive nontyphoid *Salmonella* (iNTS) and typhoid disease, and 95% Confidence Intervals (CIs) at rainfall in millimetres (mm), temperature in degrees Celsius (°C) and monthly lag-specific values relative to baseline rainfall (0 mm) and temperature (23 °C), with statistical significance (*) at p < 0.05.

	RR of iNTS, (95%CI)	RR of typhoid, (95%CI)	RR of iNTS, (95%CI)	RR of typhoid, (95%CI)
Month	Rainfall (9 mm)	Rainfall (9 mm)	Rainfall (13 mm)	Rainfall (13 mm)
Lag 0	1.20 (1.01–1.44)*	0.74 (0.46–1.19)	1.18 (0.92–1.52)	0.16 (0.06–0.52)*
Lag 1	1.28 (1.14–1.43)*	1.13 (0.80–1.59)	1.06 (0.89–1.27)	0.20 (0.10–0.43)*
Lag 2	1.32 (1.20–1.46)*	1.56 (1.21–2.02)*	0.97 (0.81–1.16)	0.26 (0.13–0.52)*
Lag 3	1.31 (1.17–1.45)*	1.86 (1.51–2.30)*	0.90 (0.73–1.10)	0.34 (0.16–0.69)*
Lag 4	1.24 (1.11–1.37)*	1.94 (1.60–2.35)*	0.85 (0.69–1.04)	0.43 (0.21–0.87)*
Lag 5	1.14 (1.03–1.26)*	1.82 (1.51–2.20)*	0.81 (0.67–0.97)*	0.55 (0.29–1.04)
Lag 6	1.02 (0.92–1.15)	1.58 (1.29–1.94)*	0.78 (0.65–0.93)*	0.69 (0.38–1.25)
Lag 7	0.91 (0.79–1.04)	1.30 (1.02–1.66)*	0.75 (0.61–0.94)*	0.87 (0.47–1.63)
Lag 8	0.79 (0.66–0.96)*	1.04 (0.76–1.42)	0.73 (0.55–0.98)*	1.1 (0.51–2.38)
Month	Temperature (19 °C)	Temperature (19 °C)	Temperature (29 °C)	Temperature (25 °C)
Lag 0	0.87 (0.76–1.01)	1.31 (0.95–1.81)	0.93 (0.73–1.20)	0.74 (0.53–1.03)
Lag 1	0.98 (0.88–1.09)	1.41 (1.09–1.84)*	0.88 (0.76–1.03)	0.93 (0.73–1.19)
Lag 2	1.07 (0.97–1.18)	1.47 (1.08–2.01)*	0.83 (0.73–0.96)*	1.12 (0.87–1.43)
Lag 3	1.11 (1.00–1.23)*	1.44 (1.02–2.05)*	0.80 (0.69–0.93)*	1.26 (0.96–1.65)
Lag 4	1.12 (1.02–1.23)*	1.35 (0.96–1.91)	0.77 (0.66–0.89)*	1.34 ((1.03–1.75*
Lag 5	1.09 (1.00–1.19)*	1.22 (0.89–1.67)	0.74 (0.65–0.84)*	1.36 (1.08–1.73)*
Lag 6	1.04 (0.95–1.13)	1.06 (0.77–1.46)	0.71 (0.62–0.82)*	1.34 (1.10–1.64)*
Lag 7	0.98 (0.88–1.08)	0.91 (0.61–1.35)	0.69 (0.56–0.84)*	1.29 (1.07–1.57)*
Lag 8	0.91 (0.80–1.04)	0.77 (0.45–1.31)	0.66 (0.50–0.88)*	1.23 (0.97–1.57)

### Comparing the delayed effects of temperature on iNTS and typhoid

**Figure 5:**
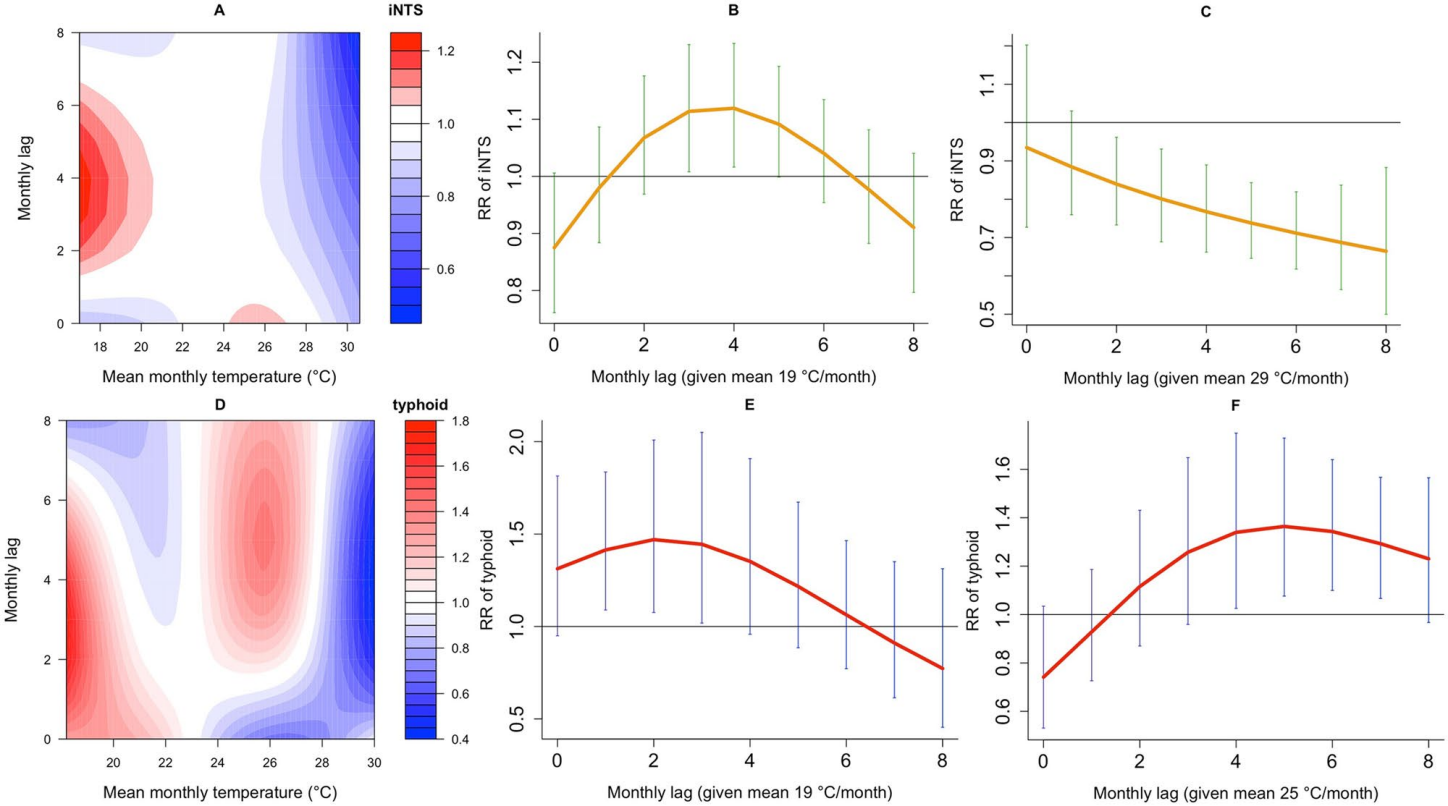
Relative risk (RR) of invasive *Salmonella* diseases, given temperature exposures and monthly lags. Contour plot of temperature-lag-nontyphoid (iNTS) (**A**) relative to baseline mean monthly temperature conditions of 23 °C at lags between 0 and 8 months, and lag-iNTS curve plots at 19 °C (**B**) and 29 °C (**C**) of temperature with 95% Confidence Intervals; Contour plot of temperature-lag-typhoid (**D**) relative to baseline mean monthly temperature conditions of 23 °C at lags between 0 and 8 months, and lag-typhoid curve plots at 19 °C (**E**) and 25 °C (**F**) of temperature with 95% Confidence Intervals.

Further, the iNTS temperature-lag relationship shows that, compared to 23 °C reference mean monthly temperature, 19 °C has a significant increased effect on the relative risk of iNTS from lags 3–5 months, peaking at lag 4 (RR 1.12, 95%CI [1.02–1.23]). For typhoid, by contrast, the temperature-lag relationship shows a bimodal pattern with increased risk at both lower (19 °C) and higher (25 °C) temperatures compared to the reference (23 °C), possibly pointing to short or long transmission cycles becoming more and less important at different temperatures. At lower temperatures, there seems to be nearly immediate increase in the risk from lags 1 to 3 months, peaking at lag 2 (RR 1.47, 95%CI [1.08–2.01]), while at higher temperatures there is a delayed and significant increased effect from lags 4–7 months, peaking at lag 5 (RR 1.36, 95%CI [1.08–1.73]). Extremely hot temperatures (>28 °C) are associated with lower incidences for both forms of invasive *Salmonella* disease (Fig. 5, and Table 1).

### Sensitivity analysis

Given the complexities of the models used and the number of parameters that could affect the results, we conducted a sensitivity analysis to assess the impact of changes in *df* for the spline functions used on the inference we draw from the models. Fixing our comparison units for mean monthly rainfall (9 mm vs. 0 mm) and temperature (19 °C vs. 23 °C) on the relative risk of iNTS, increasing the *df* in the lag space produces less smooth rainfall and temperature curves possibly indicating overfitting whereas increasing the *df* in the rainfall space produces more smooth rainfall and temperature curves suggesting controlled bias-variance tradeoff. While increasing the *df* in the temperature space does not change the rainfall curves, it produces more smooth temperature curves. (Supplementary Fig. S5). On the other hand, fixing similar comparison exposure units on the relative risk of typhoid shows that increasing the *df* in the lag or rainfall spaces profoundly reduces smoothing for rainfall and temperature curves. Counter-intuitively, increasing the *df* in the temperature space increases the precision of temperature curves especially at longer lags (Supplementary Fig. S6). Irrespective of our models’ sensitivity to changes in the *df*, the overall inferences from the models are largely unaffected, and our final models are selected based on better out-of-sample performance to achieve wider generalizability of results.

## Discussion

We empirically report on 15-year seasonal dynamics of climate and invasive *Salmonella* diseases, and described distinct and different relationships with rainfall and temperature for both forms of invasive *Salmonella* disease, by monthly lags. While an increased relative risk of iNTS was associated with lower temperatures there was a strikingly reduced risk of iNTS at extreme temperature. Typhoid, in contrast, had a bimodal pattern of increased relative-risks at both lower and higher temperatures, but with similar reduced risk occurring at extreme temperature. After the onset of moderate rainfall, the increased relative risk of iNTS was short-lasting and immediate, whereas that of typhoid was long-lasting but with a 2–4 months delay. And finally, the highest rainfalls were associated with reduced relative risk for both forms of invasive *Salmonella* disease.

In agreement with other studies^54,55^, we show that the effects of rainfall and temperature dynamics on the risk of invasive *Salmonella* diseases could be estimated in DLNM framework. Poor infrastructure and high poverty rate in Blantyre constrain access to clean water and adequate sanitation for a large number of dwellers, and particularly poorer residents, rendering the city vulnerable to climate events and their consequences for sanitation^29^. Moreover, with the growing urbanisation^56^, high-density slums are likely to expand and will present fertile ground for both short-cycle (within-household) and long-cycle (environmental) transmission routes of *Salmonella*.

Our exploratory analysis was focused around the peak of cases of iNTS between 2002 and 2003, which have been closely linked, temporally and mechanistically, to widespread *S*. Typhimurium and *S*. Enteritidis drug resistant serovars^17–19,41,57^. Comparably, the elevated cases of typhoid between 2013 and 2014 have also been attributed to the emergence of drug resistance H58 haplotype strain^13,16^. Since immune fluctuations, due to outbreaks by resistance strains or seasonal malaria/malnutrition^8^, may induce changes in susceptible population^58^, with a potential to bias the effect of climate on invasive *salmonella* diseases, control of seasonality and long-term trends reduces this bias.

This is an observational study which can only provide hypotheses of mechanism of *Salmonella* transmission dynamics. Rainfall and temperature show divergent temporal patterns across the three main seasons seen in Malawi (as described in methods), but are clearly likely to be related to each other through regional climatic weather systems. Furthermore, there are a large number of biological, environmental and social behavioural influences that might work independently or in combination to explain the relationships of both forms of invasive *Salmonella* disease that we have observed with rainfall and temperature.

One important future utility of our model could be to help predict and respond to changes in disease incidence that could follow changing regional weather patterns. For example, Southern Malawi was affected by Cyclone Idai experiencing extreme rainfall in March 2019^59^, and our model might predict that, in contrast to waterborne diseases such as cholera, excessive rainfall might not be associated with increased risk of invasive *Salmonella* diseases, which could help to inform medical responses to such climatic emergencies. Combining findings from our approach with other risk-based local or global approaches could prove particularly valuable^4,60^.

The predictions from this model may, however, also allow the generation of mechanistic hypotheses that could be prospectively tested, and used to develop specific preventive strategies. For example, our model’s estimates of an immediate and augmented effect of rainfall on the relative risk of iNTS with shorter lag times might mirror a shorter clinical incubation period for NTS strains, together with a lesser ability of NTS to survive in environmental conditions compared to *S*. Typhi bacteria^61^. This could be coupled with predominantly short-cycle transmission for iNTS within the household, perhaps when most individuals are likely to spend time indoors at the onset of rains. In contrast, the more delayed effects of rainfall on typhoid, may reflect slower clinical course and incubation period of typhoid^62^ combined with and the greater ability of the bacteria to survive and replicate in water reservoirs in the broader environment^13,63^, and a greater role of long-cycle transmission outside the household through human behaviour such as seasonal crops and harvesting activities, day care and school attendance^64^.

Interestingly, the relative-risk function of temperature for typhoid showed higher risk at both lower (an immediate effect) and higher (with a lag of 4 months or more) temperatures. This seasonal bimodal temperature-related pattern could similarly reflect the known role of the two different short and long transmission cycles for typhoid. The observed associations between extreme rainfall and highest temperature and reduced incidence of any form of invasive *Salmonella* diseases may suggest two mechanisms; surface runoff during the heaviest rains may have a consequence of reducing the environmental reservoir of *Salmonella*; and the hottest temperatures (>28 °C) may counteract the ability of bacteria to survive and multiply within households or in the environment^29^. Interestingly, our results on the effects of extreme rainfall and temperature also contradict a study in Bangladesh which reported increased risks of typhoid^63^, emphasising the likely importance of site-specific factors.

It is particularly important to note that while typhoid occurs among healthy individuals, and HIV exerts a modest protective effect against typhoid fever, there are well-established underlying risk factors for iNTS disease in adults (advanced HIV disease) and children (HIV, malaria and malnutrition)^7^. Malaria would be expected to be linked to rainfall, while malnutrition would be expected to be related to both rainfall and temperature, through an impact on crop yield and food availability. HIV, being a chronic disease, does not have a seasonal pattern, and would not be expected to have a strong influence in this model. We have previously explored and demonstrated the year-on-year relationships between iNTS disease, malaria and malnutrition using Structural Equation Modelling^8^, which suggested that at least some of the contribution of rainfall to iNTS disease might be mediated though its effects on malaria and malnutrition. These differing susceptibility factors could introduce substantial and differential time-lags into the relationships of typhoid and iNTS to climatic conditions.

This is the first study that simultaneously compares the delayed effects of rainfall and temperature on the relative risk of both iNTS and typhoid in the same setting. Our model’s typhoid risk estimates on the rainfall-lag space are consistent with findings from a previous study in the same setting^13^. However, while we observe significant lag-specific effects of climate on invasive *Salmonella* diseases, such effects have occurred at different lags and magnitude in other studies^29,30,63^, suggesting that other setting-specific factors such as elevation and hygiene may still play a crucial role in transmission pathways^2^.

Thus, in addition to possible predictive uses, and specific mechanistic hypotheses generated, our model’s suggestions may need to be verified in prospectively in Malawi using other methods, which could lead to future specific, testable, preventive interventions. Overall, the divergence of the different patterns we have observed between typhoid and iNTS disease suggest that distinctly different preventive measures against each form of *Salmonella* may be required. Other future work might also test this modelling approach in other African or global sites, where the relationship between rainfall and temperature may vary, and where the social, economic, behavioural and environmental setting is different.

The strengths of our study included the fact that both forms of invasive *Salmonella* diseases were observed at the same geographical site over a long period of time, making direct comparisons uniquely possible. Other methodological strengths include using the DLNM framework to flexibly describe nonlinear relationship between climate and invasive *Salmonella* diseases, a rigorous process of model selection by comparing Q-AIC values of fitted models, and seasonal and long-term trend adjustments to control for known and unknown confounders. Some limitations of the study also need to be highlighted. We have only reported a minimum monthly incidence of disease, and several factors likely contributed to overall under-ascertainment of invasive *Salmonella* disease. These include the fact that blood culture provides a definitive diagnosis but has a relatively low sensitivity^65^. In addition, blood cultures were only taken at the secondary hospital referral centre, and some cases may have self-medicated from community pharmacies or attended local health centres. These are, however, all likely to pertain equally throughout the year, meaning that they would have little effect on the temporal patterns used for our modelling approach. Our 15-years of surveillance did not capture spatial locations of cases making it impossible to account for spatial variability. And lastly, our models did not account for possible interactions between rainfall and temperature, or interactions with related underlying medical conditions such as human immunodeficiency virus, malaria or malnutrition.

In conclusion, we have identified, at the same geographical site, the delayed and nonlinear relationships of climate with the two forms of invasive *Salmonella* diseases commonly found in Africa, namely typhoid and iNTS disease. Our model may be useful either for surveillance, prediction and planning, or for generating and evaluating specific intervention measures. We demonstrated distinct patterns, both of lag-times and in the pattern of relationships to temperature and rainfall, suggesting that the epidemiology of iNTS disease and typhoid may also be very distinct, and this may be attributable to diverse biological, environmental, social and behavioural factors. Optimal control measures may, thus, not be the same for these two common forms of invasive *Salmonella* disease in Africa, and distinct interventions are likely to be required for disease control or prevention.

## Supplementary information

Supplementary information.

## Data Availability

A reproducible R script used to analyse the datasets is available in the *GitHub* repository; https://github.com/deusthindwa/dlnm.typhoid.nts.climate.blantyre.malawi.git.
