# Supplementary material for: Distinct climate influences on the risk of typhoid compared to invasive non-typhoid Salmonella disease in Blantyre, Malawi
Source: Sci Rep. 2019 Dec 30;9:20310. doi: 10.1038/s41598-019-56688-1 (PMC6937328; doi:10.1038/s41598-019-56688-1)
Supplement: Supplementary file 1 — Supplementary information. [file 41598_2019_56688_MOESM1_ESM.pdf]

Distinct climate influences on the risk of typhoid compared to invasive non-typhoid *Salmonella* disease in Blantyre, Malawi.

Authors: Deus Thindwa[1,2,*], Michael G. Chipeta[1,3,6], Marc Y.R. Henrion[1,4], Melita A. Gordon[1,5,6].

Affiliations

[1] Malawi-Liverpool-Wellcome Trust Clinical Research Programme, Blantyre, Malawi.

[2] Centre for Mathematical Modelling of Infectious Diseases, Department of Infectious Disease Epidemiology, London School of Hygiene and Tropical Medicine, London, United Kingdom.

[3] Nuffield Department of Medicine, Big Data Institute, Oxford University, Oxford, United Kingdom.

[4] Department of Clinical Sciences, Liverpool School of Tropical Medicine, Liverpool, United Kingdom.

[5] Institute of Infection and Global Health, University of Liverpool, Liverpool, United Kingdom.

[6] Malawi College of Medicine, University of Malawi, Blantyre, Malawi.

[*] Email: deus.thindwa@gmail.com

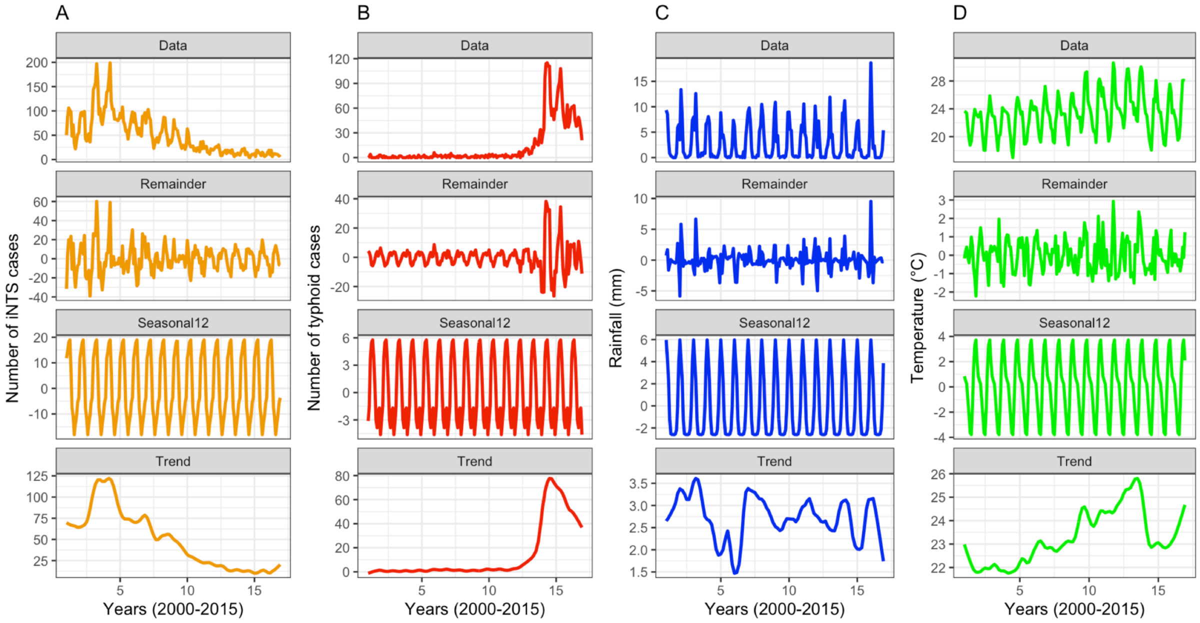

Supplementary Figure S1. Decomposition of monthly time series from years 2000 to 2015. Invasive nontyphoid *Salmonella* (iNTS) (A), typhoid (B), rainfall (C), and temperature (D) time series data from Blantyre, Malawi are decomposed into remainder, monthly seasonal, and trend components separately.

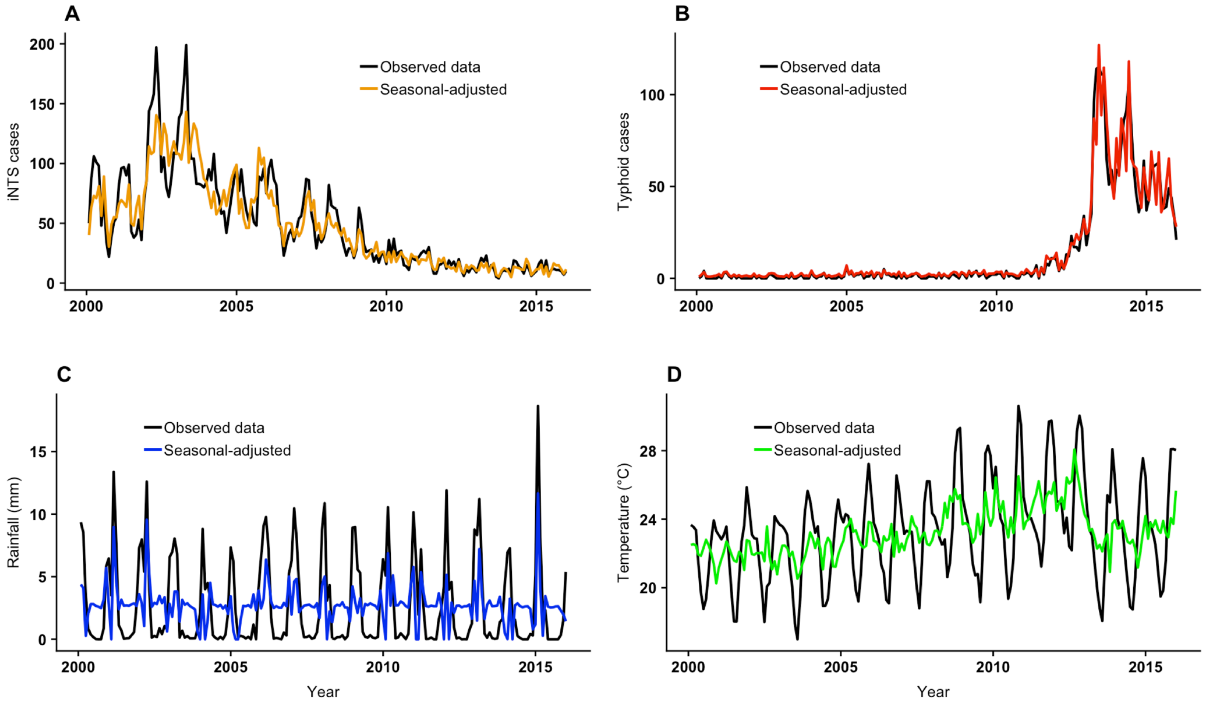

Supplementary Figure S2. Seasonal-unadjusted versus seasonal-adjusted monthly time series. Invasive nontyphoid (iNTS) (A), typhoid (B), rainfall (C), and temperature (D) time series data from Blantyre, Malawi over a period of 16 years.

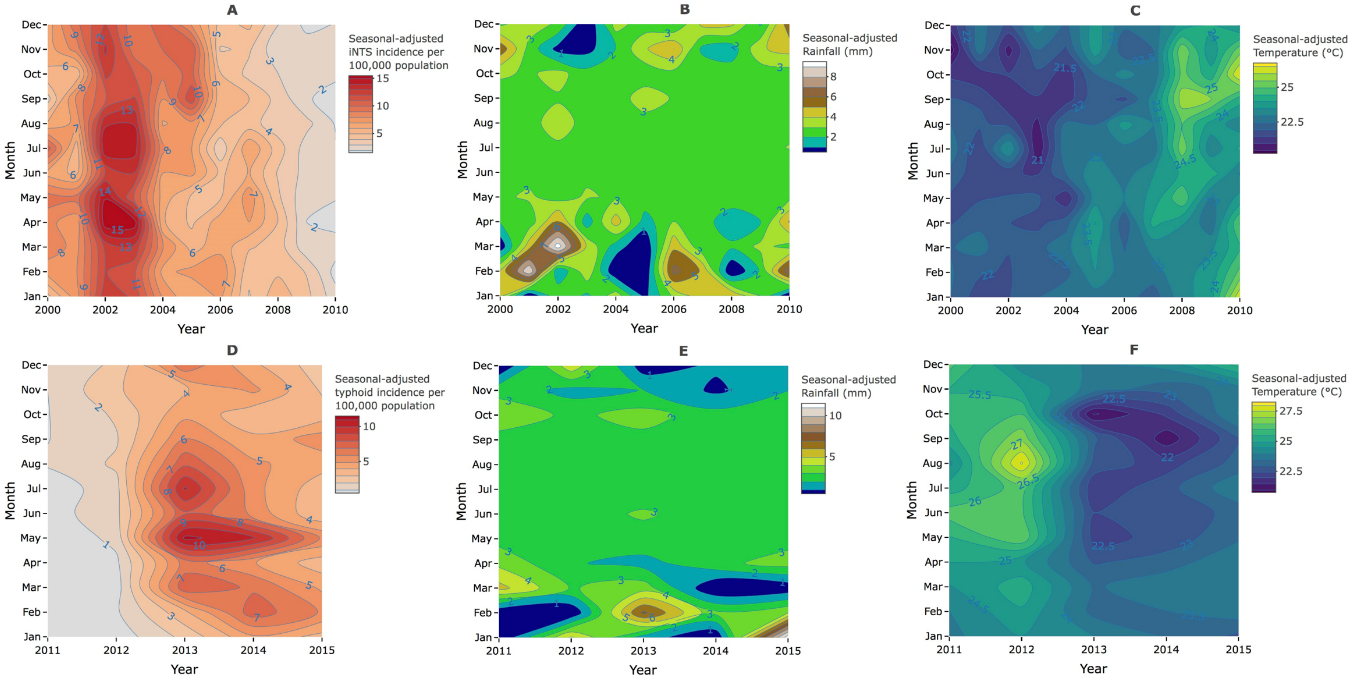

Supplementary Figure S3. Seasonal-adjusted annual cycle of *Salmonella* incidence rates and climate. Dynamics of invasive nontyphoid *Salmonella* incidence (iNTS) (A), rainfall (B) temperature (C) over 11 years (between years 2000 and 2010), and typhoid cases (D), rainfall (E) and temperature (F) over 5 years (between years 2011 and 2015) following monthly seasonal-adjustment using time series data from Blantyre, Malawi.

Model	$w(l)$	$f(x_1)$	$f(x_2)$	$iNTS_R$	$iNTS_T$	iNTS	$Typhoid_R$	$Typhoid_T$	Typhoid
#	*df*	*df*	*df*	*Q-AIC*	*Q-AIC*	*Q-AIC*	*Q-AIC*	*Q-AIC*	*Q-AIC*
1	3	3	3	570.7089	580.4330	567.9318*	212.1887	222.8303	210.6648*
2	4	3	3	574.2819	585.1239	578.3531	217.4634	212.1270	217.8383
3	5	3	3	580.2446	589.3765	587.3423	222.4061	216.4683	228.4523
4	3	4	3	574.4757	580.4330	572.1161	204.9102*	222.8303	214.0661
5	4	4	3	579.8267	585.1239	584.5922	212.4587	212.1270	224.8071
6	5	4	3	587.5728	589.3765	595.1572	216.0560	216.4683	236.6332
7	3	5	3	573.9352	580.4330	568.5534	209.8391	222.8303	217.8914
8	4	5	3	580.8374	585.1239	583.1928	216.7759	212.1270	230.4198
9	5	5	3	590.4370	589.3765	594.1826	222.3633	216.4683	245.0488
10	3	3	4	570.7089	585.5078	570.8126	212.1887	226.2686	212.3317
11	4	3	4	574.2819	588.9740	582.0334	217.4634	219.7139	223.4347
12	5	3	4	580.2446	587.7656	591.6384	222.4061	224.9809	236.3496
13	3	4	4	574.4757	585.5078	573.3518	204.9102	226.2686	217.9361
14	4	4	4	579.8267	588.9740	587.5944	212.4587	219.7139	231.2642
15	5	4	4	587.5728	587.7656	600.0265	216.0560	224.9809	245.6914
16	3	5	4	587.5728	585.5078	573.7475	209.8391	226.2686	221.1059
17	4	5	4	580.8374	588.9740	590.1806	216.7759	219.7139	237.9325
18	5	5	4	590.4370	587.7656	602.4179	222.3633	224.9809	253.1268
19	3	3	5	570.7089	584.5078	571.3729	212.1887	218.5320	217.8403
20	4	3	5	574.2819	592.4074	582.8894	217.4634	213.9474	231.1740
21	5	3	5	580.2446	590.6842	594.6393	222.4061	222.1824	245.2023
22	3	4	5	574.4757	584.5078	575.8213	204.9102	218.5320	223.3430
23	4	4	5	579.8267	592.4074	590.1457	212.4587	213.9474	238.9668
24	5	4	5	587.5728	590.6842	603.8391	216.0560	222.1824	254.4714
25	3	5	5	573.9352	584.5078	578.8830	209.8391	218.5320	227.2069
26	4	5	5	580.8374	592.4074	595.8418	216.7759	213.9474	245.8361
27	5	5	5	590.4370	590.6842	610.4309	222.3633	222.1824	260.6858

Supplementary Table S1. Natural cubic spline functions for monthly rainfall $f(x_1)$, monthly temperature $f(x_2)$ and monthly lag $w(l)$. Individual degrees of freedom (*df*) associated with cross-basis matrix in the exposure-lag space, and values for the Quasi-Akaike Information Criterion (Q-AIC) for the alternative models for the association between invasive nontyphoid disease and rainfall ($iNTS_R$) or temperature ($iNTS_T$) or both (iNTS) from year 2000 to 2010, and for the association between typhoid and rainfall ($Typhoid_R$) or temperature ($Typhoid_T$) or both (Typhoid) from year

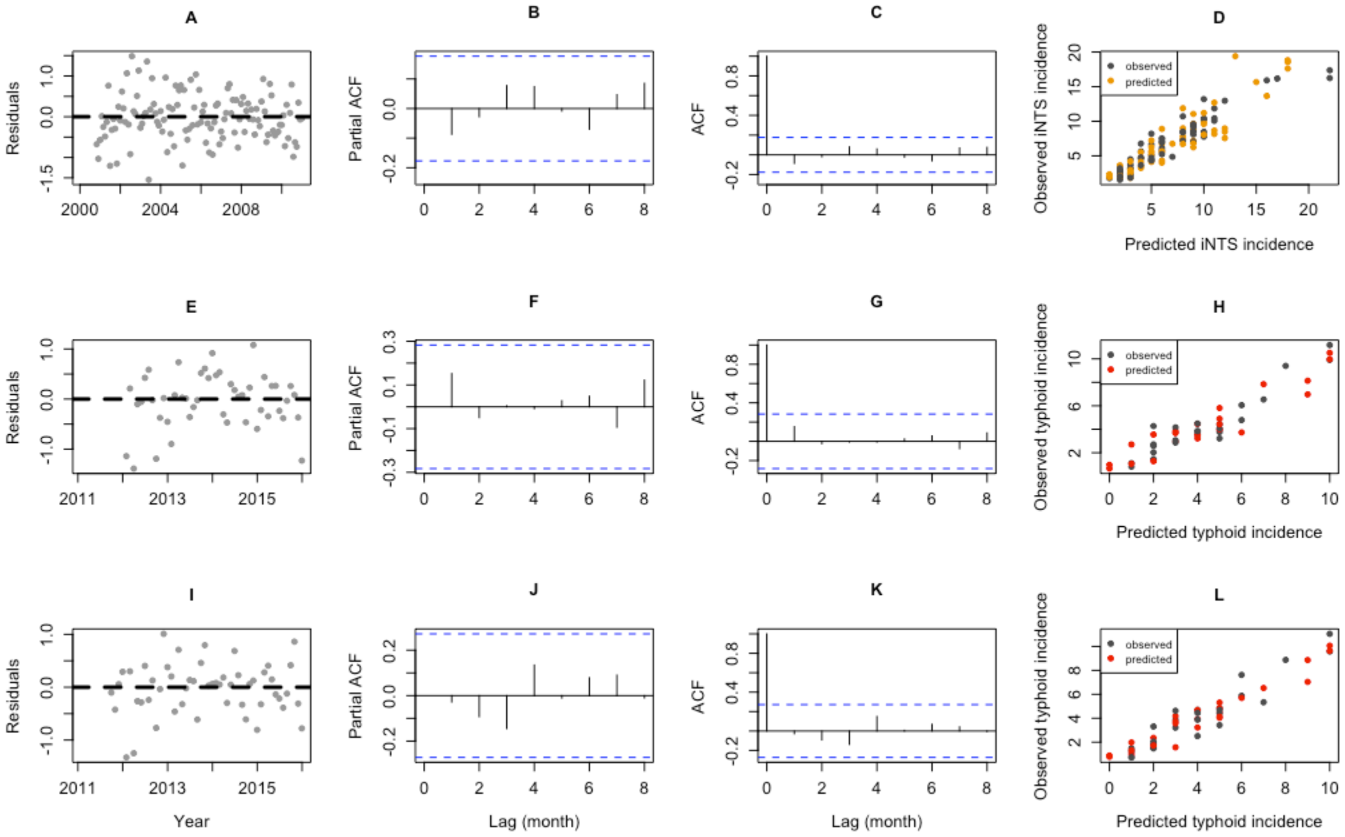

Supplementary Figure S4. Model validation checks. Deviance residuals overtime, partial autocorrelation (partial ACF), autocorrelation (ACF) and model predictions respectively, estimated by; adjusted model (additional residuals at lag 1) of bi-dimensional association between rainfall-lag and temperature-lag and invasive nontyphoid *Salmonella* (iNTS) (A,B,C,D), adjusted model (additional residuals at lag 4) of bi-dimensional association between rainfall-lag and typhoid (E,F,G,H), and original model of bi-dimensional association between temperature-lag and rainfall-lag and typhoid (I,J,K,L).

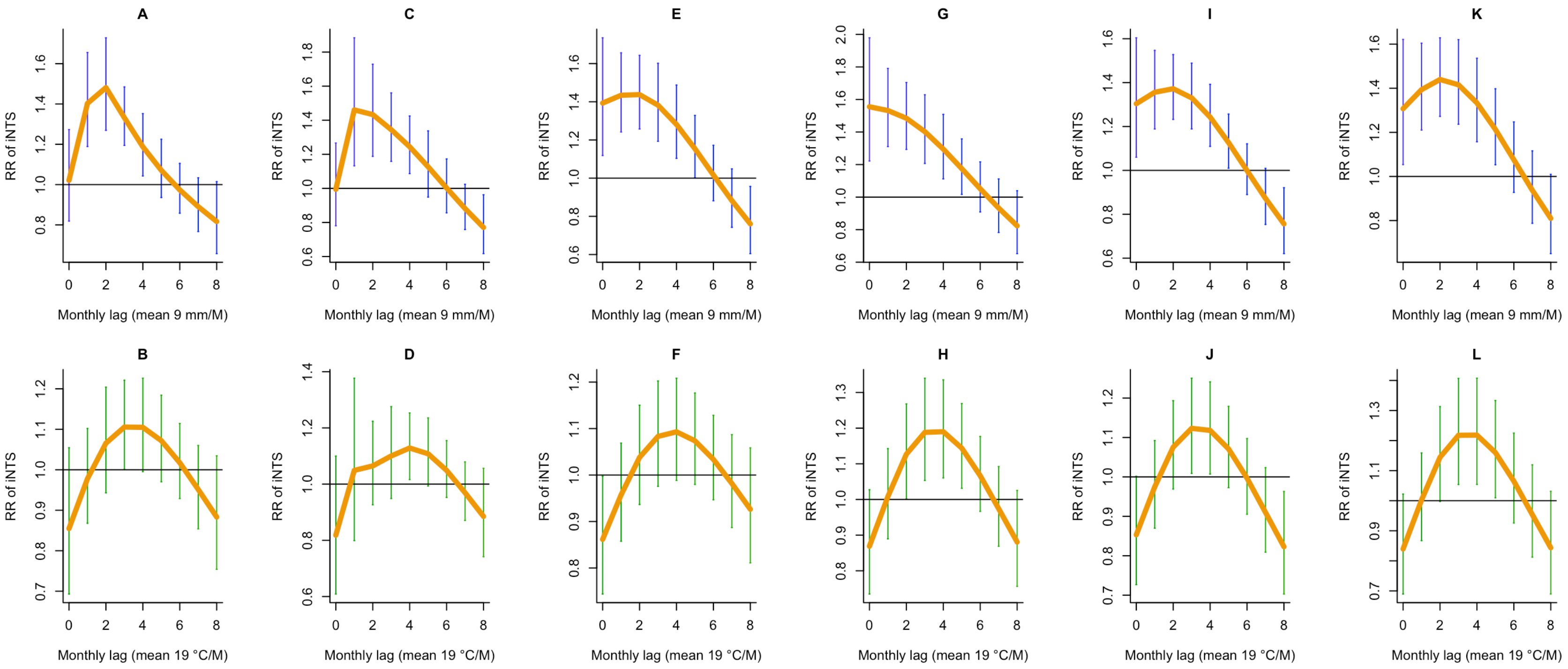

Supplementary Figure S5. Sensitivity analysis. The effects on invasive nontyphoid *Salmonella* (iNTS) of mean 9 mm/month (mm/M) rainfall relative to 0 mm and mean 19 °C/month (°C /M) temperature relative to 23 °C along the lags, varied by increasing degrees of freedom (*df*) in the lag space from (*df*=4; model 2; A,B) to (*df*=5; model 3; C,D), in the rainfall space from (*df*=4; model 4; E,F) to (*df*=5; model 7; G,H), and in the temperature space from (*df*=4; model 10; I,J) to (*df*=5; model 19; K,L).

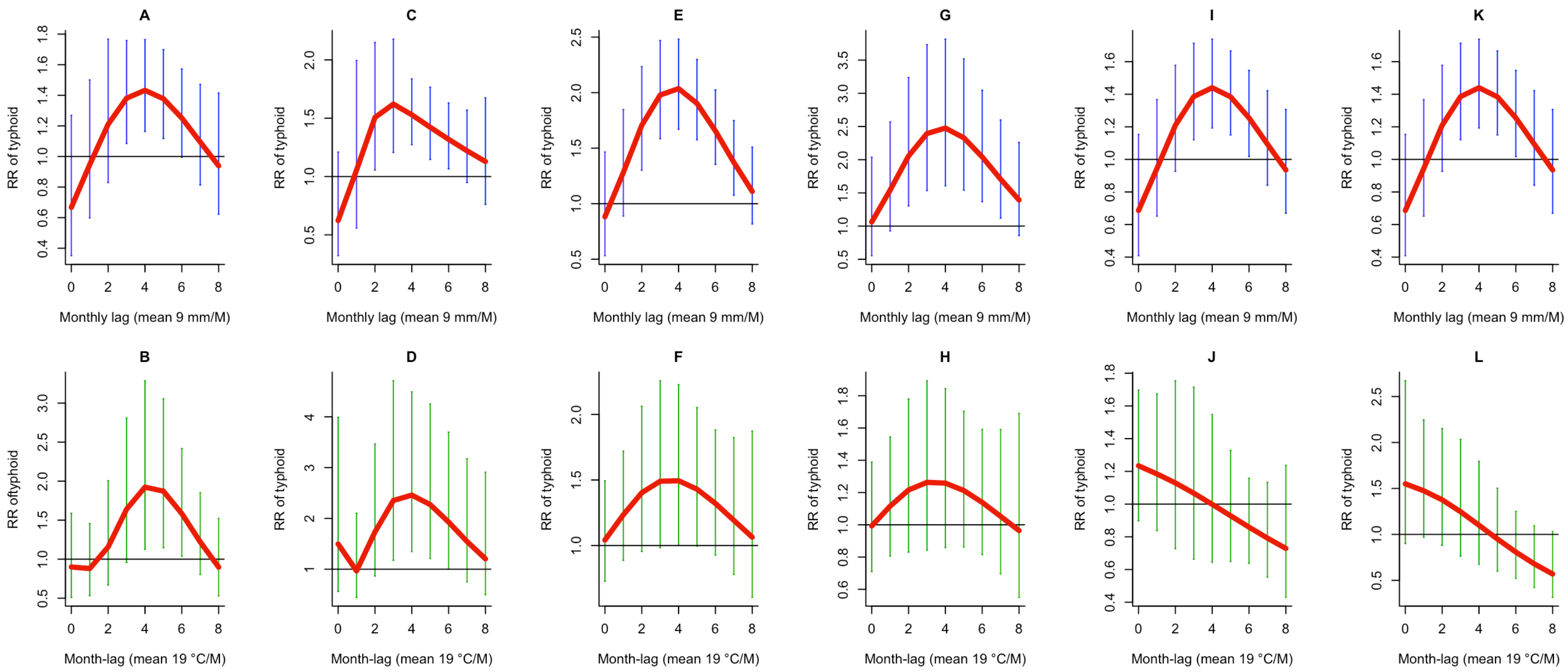

Supplementary Figure S6. Sensitivity analysis. The effects on typhoid of mean 9 mm/month (mm/M) rainfall relative to 0 mm and mean 19 °C/month (°C/M) temperature relative to 23 °C along the lags, varied by increasing the degree of freedom (*df*) in the lag space from (*df*=4; model 2; A,B) to (*df*=5; model 3; C,D), in the rainfall space from (*df*=4; model 4; E,F) to (*df*=5; model 7; G,H), and in the temperature space from (*df*=4; model 10; I,J) to (*df*=5; model 19; K,L).

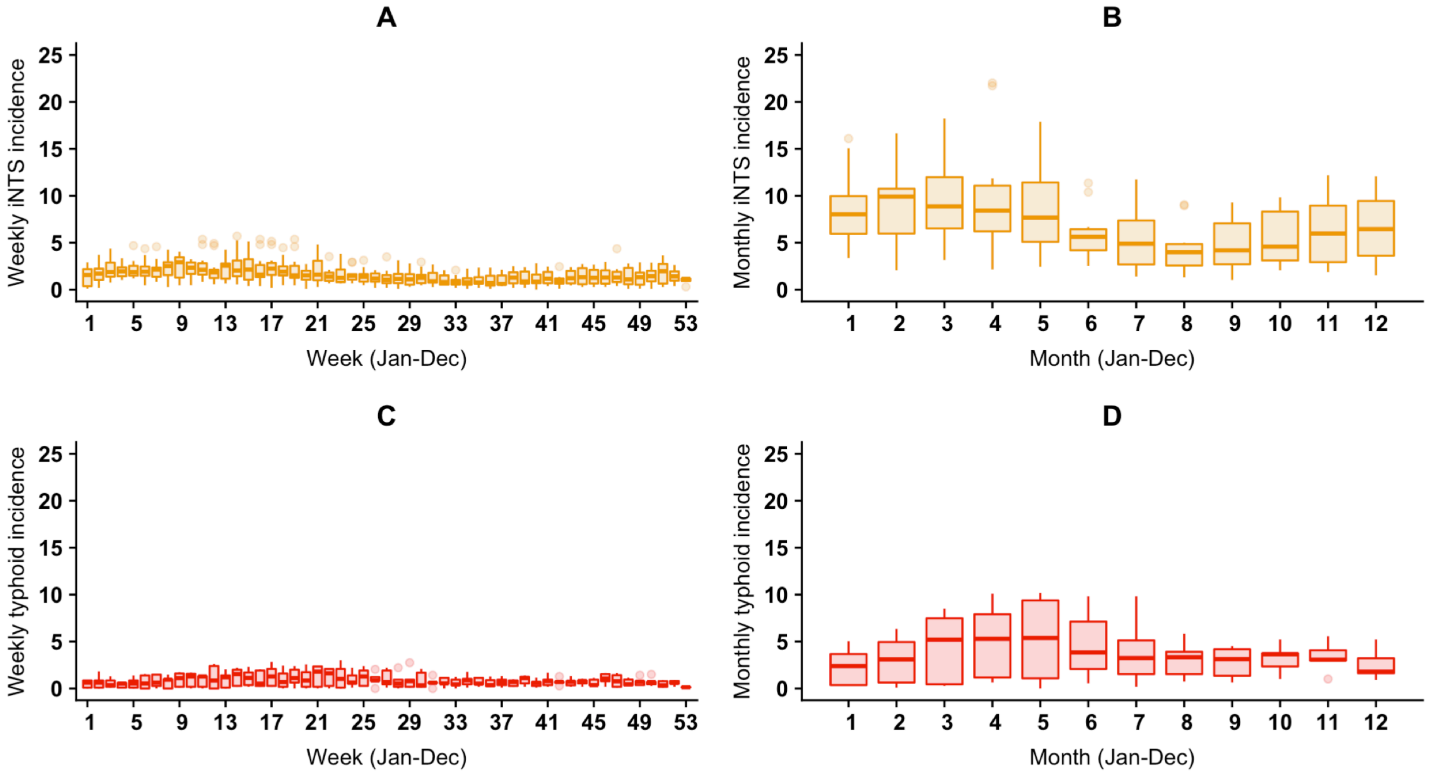

Supplementary Figure S7. Weekly versus monthly seasonal-unadjusted incidence of invasive *Salmonella* diseases. Weekly and monthly incidence of invasive nontyphoid *Salmonella* over 11 years (A and B) and typhoid over 5 years (C and D), respectively.

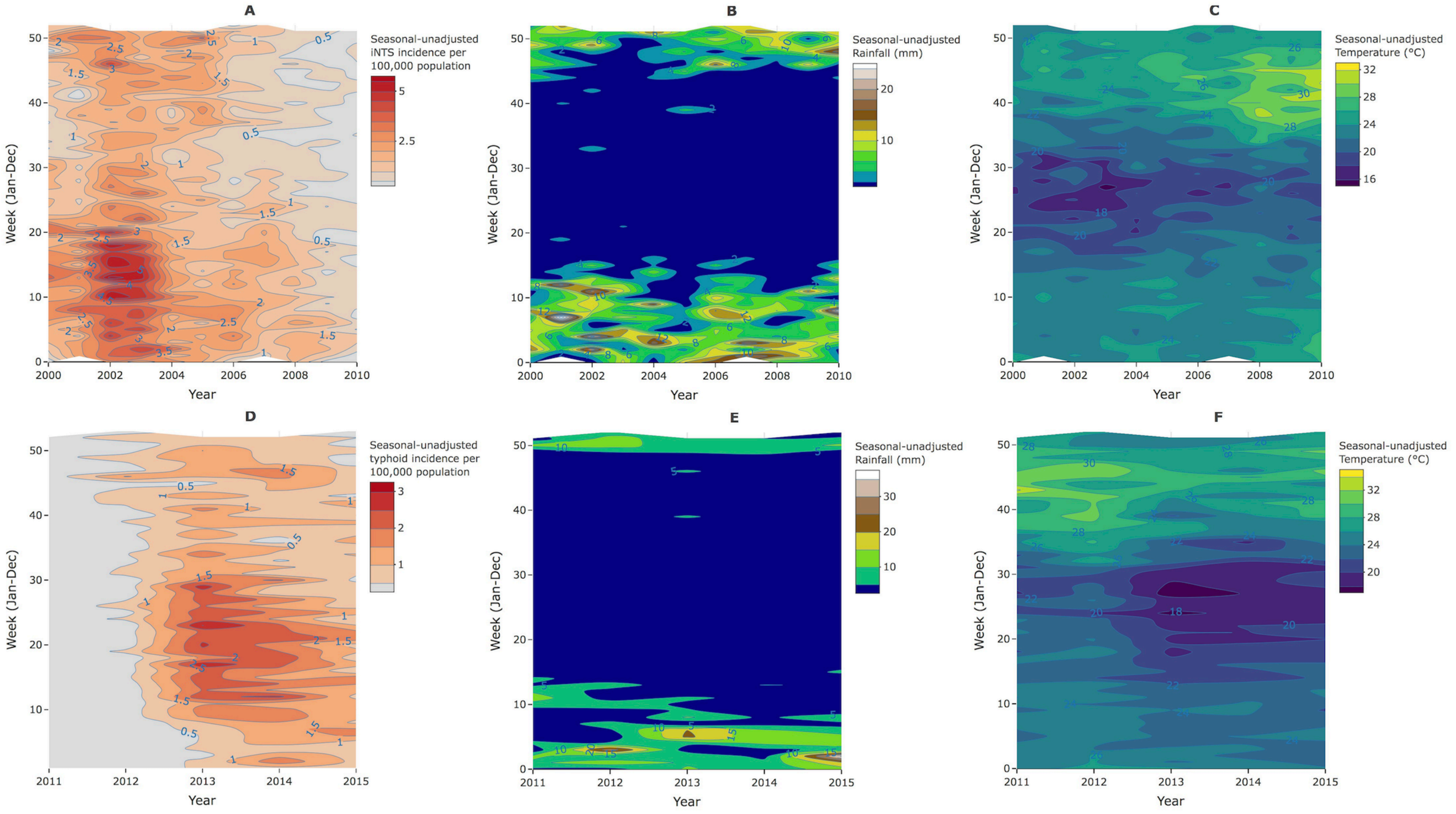

Supplementary Figure S8. Weekly seasonal-unadjusted annual cycle of *Salmonella* incidence rates and climate. Weekly dynamics of invasive nontyphoid *Salmonella* incidence (iNTS) (A), rainfall (B) temperature (C) over 11 years (between years 2000 and 2010), and typhoid cases (D), rainfall (E) and temperature (F) over 5 years (between years 2011 and 2015) using time series data from Blantyre, Malawi.

model.no	lag.df	fx1.df	fx2.df	QAIC.ntsR	QAIC.ntsT	QAIC.nts	QAIC.typR	QAIC.typT	QAIC.typ
1	3	3	3	1524.757	1519.700	1531.143	540.9125	531.7717	545.3841
2	4	3	3	1530.390	1522.270	1539.625	546.5628	536.6449	555.6807
3	5	3	3	1535.525	1525.789	1548.621	552.1588	542.4188	566.6796
4	3	4	3	1530.054	1519.700	1536.337	546.8450	531.7717	551.4437
5	4	4	3	1536.947	1522.270	1546.137	554.3891	536.6449	563.5952
6	5	4	3	1544.099	1525.789	1557.163	562.0286	542.4188	576.5845
7	3	5	3	1534.200	1519.700	1539.776	544.4189	531.7717	548.4657
8	4	5	3	1541.237	1522.270	1550.055	553.8608	536.6449	562.7434
9	5	5	3	1550.509	1525.789	1563.216	563.4763	542.4188	577.7582
10	3	3	4	1524.757	1520.694	1532.983	540.9125	536.3786	548.7937
11	4	3	4	1530.390	1525.057	1543.237	546.5628	543.4552	561.3984
12	5	3	4	1535.525	1530.582	1554.033	552.1588	550.9330	574.3019
13	3	4	4	1530.054	1520.694	1538.034	546.8450	536.3786	555.0159
14	4	4	4	1536.947	1525.057	1549.584	554.3891	543.4552	569.5439
15	5	4	4	1544.099	1530.582	1562.322	562.0286	550.9330	584.4541
16	3	5	4	1534.200	1520.694	1541.870	544.4189	536.3786	551.2130
17	4	5	4	1541.237	1525.057	1553.874	553.8608	543.4552	567.8638
18	5	5	4	1550.509	1530.582	1568.705	563.4763	550.9330	584.8132
19	3	3	5	1524.757	1525.748	1538.441	540.9125	541.2480	554.7443
20	4	3	5	1530.390	1531.911	1550.446	546.5628	550.1579	569.1694
21	5	3	5	1535.525	1539.070	1562.984	552.1588	559.4527	583.7914
22	3	4	5	1530.054	1525.748	1543.752	546.8450	541.2480	560.9574
23	4	4	5	1536.947	1531.911	1557.137	554.3891	550.1579	577.3095
24	5	4	5	1544.099	1539.070	1571.667	562.0286	559.4527	593.9266
25	3	5	5	1534.200	1525.748	1547.426	544.4189	541.2480	557.3518
26	4	5	5	1541.237	1531.911	1561.154	553.8608	550.1579	575.7929
27	5	5	5	1550.509	1539.070	1577.779	563.4763	559.4527	594.2512

Supplementary Table S2. Natural cubic spline functions for weekly rainfall ($fx1.df$), weekly temperature ($fx2.df$) and weekly lag ($lag.df$). Individual degrees of freedom (*df*) associated with cross-basis matrix in the exposure-lag space, and values for the Quasi-Akaike Information Criterion (QAIC) for the alternative models for the association between invasive nontyphoid disease and rainfall (QAIC.ntsR) or temperature (QAIC.ntsT) or both (QAIC.nts) from year 2000 to 2010, and for the association between typhoid and rainfall (QAIC.typR) or temperature (QAIC.typT) or both (QAIC.typ) from year 2011-2015 in Blantyre, Malawi.

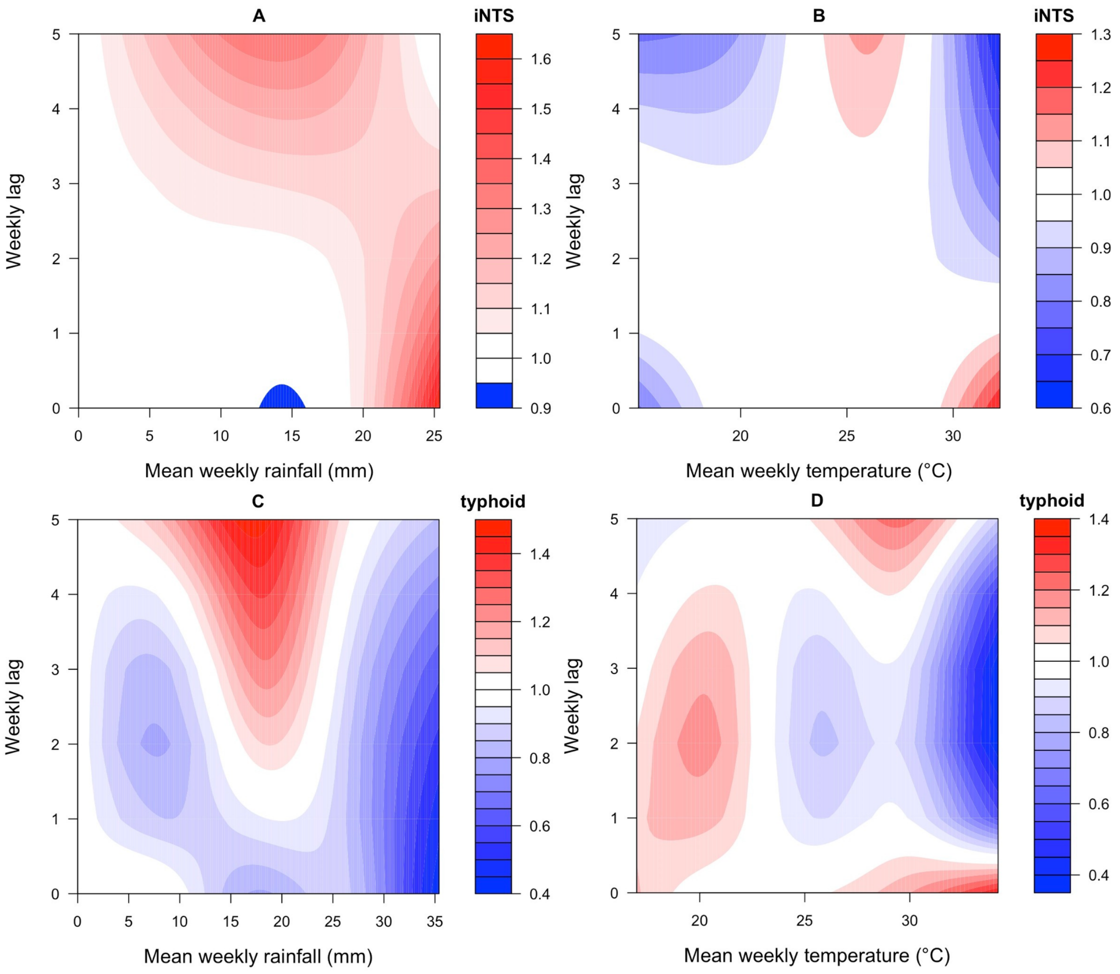

Supplementary Figure S9. Relative risk of invasive *Salmonella* diseases given rainfall and temperature exposures, and weekly lags. Contour plots of rainfall-lag-invasive nontyphoid *Salmonella* (iNTS) risk (A), temperature-lag-invasive nontyphoid *Salmonella* (iNTS) risk (B), rainfall-lag-typhoid risk (C), temperature-lag-typhoid risk (D).
